# Taro Lectin Can Act as a Cytokine-Mimetic Compound, Stimulating Myeloid and T Lymphocyte Lineages and Protecting Progenitors in Murine Bone Marrow

**DOI:** 10.3390/pharmaceutics13030350

**Published:** 2021-03-07

**Authors:** Erika Bertozzi de Aquino Mattos, Patricia Ribeiro Pereira, Lyris Anunciata Demétrio Mérida, Anna Carolina Nitzsche Teixeira Fernandes Corrêa, Maria Paula Vigna Freire, Vania Margaret Flosi Paschoalin, Gerlinde Agate Platais Brasil Teixeira, Maria de Fátima Brandão Pinho, Maurício Afonso Verícimo

**Affiliations:** 1 Biology Institute, Federal University (UFF), Rua Alexandre Moura, No. 8, Bloco M, Sala. 505, Gragoatá, Niterói, RJ 24210-200, Brazil; erika_bertozzi@hotmail.com (E.B.d.A.M.); lyrisdemetriomerida@gmail.com (L.A.D.M.); mariavfreire@gmail.com (M.P.V.F.); gerlinde.teixeira@gmail.com (G.A.P.B.T.); pmfat@hotmail.com (M.d.F.B.P.); mavericimo@gmail.com (M.A.V.); 2 Chemistry Institute, Federal University of Rio de Janeiro (UFRJ), Av. Athos da Silveira Ramos, 149, Sala 545, Cidade Universitária, Rio de Janeiro, RJ 21941-909, Brazil; patriciarp@iq.ufrj.br (P.R.P.); annac.correa@hotmail.com (A.C.N.T.F.C.)

**Keywords:** tarin, immunomodulation, hematopoietic progenitor cells, lymphocyte differentiation, granulocyte stimulation, interleukin-3-like, GM-CSF-like

## Abstract

Taro (*Colocasia esculenta*) corm is traditionally consumed as a medicinal plant to stimulate immune responses and restore a health status. Tarin, a taro lectin, is considered responsible for the immunomodulatory effects of taro. In the present study, in order to investigate the effects of tarin on bone marrow hematopoietic population, murine cells were stimulated with tarin combined with a highly enriched conditioned medium containing either IL-3 or GM-CSF. Cells challenged with tarin proliferated in a dose-dependent manner, evidenced by the increase in cell density and number of clusters and colonies. Tarin exhibited a cytokine-mimetic effect similar to IL-3 and GM-CSF, increasing granulocytic cell lineage percentages, demonstrated by an increase in the relative percentage of Gr-1+ cells. Tarin does not increase lymphocytic lineages, but phenotyping revealed that the relative percentage of CD3+ cells was increased with a concomitant decrease in CD19+ and IL-7Rα+ cells. Most bone marrow cells were stained with tarin-FITC, indicating non-selective tarin binding, a phenomenon that must still be elucidated. In conclusion, taro corms contain an immunomodulatory lectin able to boost the immune system by promoting myeloid and lymphoid hematopoietic progenitor cell proliferation and differentiation.

## 1. Introduction

Taro (*Colocasia esculenta*), an edible plant belonging to the Araceae family, is mainly consumed in underdeveloped countries as a subsistence culture and is used as a medicinal plant [1,2,3]. Taro corms are a significant source of carbohydrates, followed by dietary fibers, proteins, vitamins such as folates, niacin, pantothenic acid, pyridoxin, riboflavin, thiamin, vitamin A, C, E, and K, and minerals including calcium, copper, iron, magnesium, manganese, selenium, and zinc [4]. In addition to their nutritional value, taro corms are rich in bioactive compounds such as tarin, taro-4-I polysaccharide, TPS1/TPS2 polysaccharides, A-1/B-2 α-amylase inhibitors, monogalactosyldiacylglycerols (MGDGs), digalactosyldiacylglycerols (DGDGs), polyphenols, and nonphenolic antioxidants that are responsible for antitumoral, anti-metastatic, anti-mutagenic, immunomodulatory, anti-inflammatory, antioxidant, anti-hyperglycemic, and anti-hyperlipidemic activities, supporting their ancient application for medicinal purposes by indigenous people and, thus considered a functional food [1,5].

Widely explored, tarin, a 47 kDa heterotetramer protein present in taro corms, is a lectin belonging to the *Galanthus nivalis* agglutinin (GNA)-related superfamily with the ability to preferentially bind to mannose-based and complex N-glycans, which are typically comprised of cell antigens of both healthy and unhealthy animal tissues and microorganisms. It is widely believed that, similarly to other members of the GNA-related family, tarin-carbohydrate interactions are essential to promote main tarin bioactivities, such as the antitumoral [6,7,8], anti-insect [9,10,11,12,13], immunomodulatory [14,15], and antiviral [16] effects. In fact, taro lectin exhibits affinity to glycans that make up part of certain antigens, such as gp-120 found in HIV, CA-125 in ovarian cancer cells, the Lewis^y^ epitope, and H2 antigens found in a wide range of cancer cells, in both hematopoietic progenitors, and in peripheral blood granulocytes [17,18,19,20,21,22].

The association of antitumoral and immunomodulatory effects by a single molecule, as in tarin, can be very advantageous to potentiate chemotherapeutic treatments, stimulating studies in this field [1,14,23,24]. Tarin is effective in in vitro assays against human tumoral lineages, such as MDA-MB-231, MCF10A, and MCF-7 breast cancer lineages, the hepatoma HepG2 lineage, adult T-cell leukemia ED, Su9T01 and S1T lineages, murine breast cancer 66.1, 410.4 and EpH4 lineages, and murine melanoma B16BL6, as well as in YYT rat colon cancer cells [6,7]. Furthermore, tarin is also able to prevent lung colonization and metastasis by the 66.1 breast cancer cell lineage in murine models [6].

Tarin encapsulation in liposome nanocapsules results in improvements of its in vitro antitumoral activity against human MDA-MB-231 breast cancer and human U-87 MG glioblastoma, achieving efficiencies similar to cisplatin and temozolomide, widely applied as chemotherapeutic drugs [8].

Tarin immunomodulatory properties have been explored both in vivo and ex vivo, revealing certain particularities, such as the ability to stimulate hematopoietic cell proliferation, especially B220^+^ spleen lymphocytes. Recent studies have reported that tarin also affects the myeloid population, particularly granulocytes, both in the bone marrow and in blood circulation, with the ability to minimize leukopenia and genotoxicity in cyclophosphamide-immunosuppressed mice while accelerating animal recovery [14,15,23].

Tarin may become recognized as a novel bioactive food product from an edible tubercle with no toxic effects and stable under a wide range of pH and temperatures. It can be obtained in a pure form at a relatively low cost using a well-established and high yield purification method [14,23]. In order to prospect tarin’s pharmacological use, studies are underway to understand its antitumoral and immunomodulatory mechanisms of action, as well as their interconnections. Herein, the effects of tarin on murine bone marrow (BM) myeloid and lymphoid populations associated or not with growth factors, such as IL-3 and GM-CSF, were investigated. Tarin binding to distinct BM hematopoietic populations was also addressed by applying fluorochrome staining.

## 2. Materials and Methods

### 2.1. Animals and Growth Factors

Adult male C57BL/6 mice were provided by the Center of Laboratory Animal (NAL) belonging to the Federal Fluminense University (UFF) and were maintained at the UFF Biology Institute under conventional environmental conditions with exhaust fans, at a room temperature of 23–25 °C and fed Nuvilab CR-1 chow (Nuvital Nutrientes S/A, Colombo, BRA) and acidified water ad libitum to avoid bacteria proliferation [25,26]. The research protocol was approved by the Federal Fluminense University Animal Use Ethics Committee (CEUA), under number 670/2016.

Conditioned media enriched with interleukin-3 (cmIL-3) and granulocyte and macrophage colony-stimulating factor (cmGM-CSF) were respectively obtained from two cell lineages, WEHI-3B (ATCC TIB-68) and M3 (BCRJ code 0154), purchased from the Rio de Janeiro Cell Bank (BCRJ), RJ, Brazil.

### 2.2. Tarin Purification

*C. esculenta* (L.) Schott corms were purchased from a local market in Rio de Janeiro, Brazil (22°54′29.9988′′ S and 43°11′46.9968′′ W) and used to prepare the crude taro extract (CTE) [12]. Tarin was purified by affinity chromatography using a Cibacron Blue 3GA column (Sigma-Aldrich Co, San Luis, MO, USA) [14]. Purified tarin concentrations were estimated using bovine serum albumin (BSA) (Sigma-Aldrich Co.) as an external standard [27].

### 2.3. Mice Bone Marrow Cell Tarin-Binding Evaluations

Purified tarin was conjugated to fluorescein isothiocyanate (FITC) (Riedel-de-Haen-Cat 33225), following the manufacturer’s instructions [28,29,30]. FITC (1 mg/mL) in anhydrous DMSO was slowly added to a tarin solution (2 mg/mL) in 0.1 M Na_2_CO_3_ pH 9.0 at a 1:20 (*v/v*) ratio, following overnight incubation at 4 °C while maintaining gentle and continuous stirring. After the incubation period, NH_4_Cl was added to a final concentration of 50 mM, and the solution was kept for 2 h at 4 °C, following xylene cyanol at 0.1% and 5% glycerol addition. The tarin-FITC was purified using a gel filtration chromatography column (Bio-Gel P-30, Bio-Rad Laboratories, Hercules, CA, USA) previously equilibrated with PBS pH 7.5 at a flow rate of 9.6 mL/h. Tarin-FITC fractions with absorbances in the range of 0.2 to 1.40 at 495 nm and 280 nm using a Beckman DU 640 spectrophotometer (Beckman Coulter, Brea, CA, USA) were pooled. Tarin-FITC concentrations were estimated according to absorbances at 280 nm and stored in the dark at 4 °C until use.

Bone marrow (BM) cells (1.0 × 10^6^ cell/mL) were incubated with tarin-FITC for 60 min at 4 °C and fluorescence intensities were monitored every 10 min following the flow cytometry protocol described in Section 2.6. The control group, which consisted of BM cells that were subjected to the entire procedure but did not receive tarin-FITC, was used to exclude autofluorescence.

### 2.4. Bone Marrow Cell Suspensions and Culture Conditions

One mouse was euthanized with a lethal dose of 40 mg/kg xylazine and 200 mg/kg ketamine, with death ensured by cervical dislocation. BM cells (2 × 10^6^ cells/mL), obtained by percolating the two femurs with sterile PBS, were cultured in RPMI-1640 medium (Sigma-Aldrich Co.), supplemented with 10% (*v/v*) fetal calf serum (FCS), 2 mM l-glutamine, 5 × 10^−5^ M 2-beta-mercaptoethanol and 20 μg/mL gentamycin during 12 days at 37 °C in a humidified atmosphere containing 5% CO_2_ [31].

### 2.5. Tarin Effects on Murine Bone Marrow Cell Cultures

BM cell cultures were challenged on day 0 with a single tarin dose ranging from 6 to 100 μg/mL followed by culturing for 12 days. The number of discrete aggregates (3–50 cells scored as clusters and 50 or more cells scored as colonies) were counted in distinct fields at different timepoints. Cell densities were calculated as the percentage of occupied area/field using the ImageJ software version 2 (Wayne Rasband, Bethesda, MD, USA) [32]. Photomicrographs were obtained using an inverted-phase Zeiss Telaval microscope (model 31) (Carl Zeiss Co., Oberkochen, Germany). On the last day of culture, cells were harvested using sterile cell scrapers and transferred to glass slides by centrifugation at 284× *g* for 10 min, at room temperature, using a Cytopro 7620 centrifuge (WESCOR Inc., Logan, UT, USA). Cells were then stained following the May-Grunwald-Giemsa method [33,34], and photomicrographs of the cultures were acquired under a n optical microscope (Olympus BX41) using the Future WinJoe v1.0.7.9 software (Future Optics Sci. & Tech. Co, Xiasha, Hangzhou, China). 

### 2.6. Flow Cytometry Analysis of BM Stimulated with Tarin and/or Growth Factors: Cell Distribution and Phenotype Profile

BM cells were adjusted to 2.0 × 10^6^ in a final volume of 2 mL of RPMI. Tarin (25 µg/mL), 10% (*v/v*) conditioned interleukin-3-enriched media (cmIL-3), 10% (*v/v*) rich in granulocyte and macrophage colony-stimulating factor (cmGM-CSF), cmIL-3 associated with tarin (cmIL-3+tarin) or cmGM-CSF associated with tarin (cmGM-CSF+tarin) were used to stimulate the BM cells. Cells were harvested using sterile cell scrapers and analyzed by flow cytometry regarding their size (FSC—forward scatter) and granularity (SSC—side scatter) on the 5th day post-stimulation. Cells were then counted and, to avoid underappreciation, analyzed considering two specific regions, gate A, corresponding to higher granularity cells like granulocytes, and gate B, corresponding to lower granularity cells, such as lymphocyte and progenitors. Flow cytometry data were acquired and analyzed on a BD Accuri C6 Flow Cytometer (BD Bioscience, San Jose, CA, USA). Data were analyzed using the BD Accuri C6 software and expressed as percentages.

On the 5th day post-stimulation, when cell profiles greatly differ from the control, and a strong repopulation/proliferation/maintenance response in tarin-stimulated group is detected [15], bone marrow cell cultures were harvested using sterile cell scrapers and stained with molecular markers to evaluate the effect of tarin and/or growth factors on myeloid (Gr-1) and lymphoid (CD3, CD19 and IL-7Ra) cell populations. Cell suspensions (1.0 × 10^6^ cells) were incubated with 200 μL of a blocking solution (PBS containing 3% FCS + 10% control mouse serum + 0.001% sodium azide) for 15 min at 4 °C to prevent non-specific antibody binding. After incubation, cells were washed in PBS and recovered by centrifugation at 200× *g* for 10 min at 4 °C followed by incubation with anti-CD3 FITC, anti-CD19 PE, biotinylated anti-Gr-1 (Ly-6G/Ly-6C) or biotinylated anti-IL-7Rα (Bio Legend Inc., San Diego, CA, USA) for 30 min at 4 °C. Biotinylated primary antibodies were coupled to streptavidin allophycocyanin (SAV-APC) (Bio Legend Inc.) for 30 min at 4 °C. Antibodies and biomarkers were dissolved in PBS containing 3% FCS, 10% control mouse serum, and 0.001% sodium azide. Cells were washed by centrifugation at 200× *g* for 7 min at 4 °C and subsequently fixed in 200 μL of PBS containing 1% formaldehyde for acquisition on the BD Accuri C6 Flow Cytometer (BD Bioscience), using the BD Accuri C6 software (BD Bioscience).

### 2.7. Statistical Analyses

All data are expressed as means ± standard deviations (SD), using the GraphPad Prism v.7 software, considering *p* < 0.05 as significant. Statistically significant differences were assessed using Student’s *t*-test, or one-way ANOVA with Tukey’s post-hoc test for multiple comparisons.

## 3. Results

### 3.1. Selective Tarin Binding to a Bone Marrow Cell Population

**Figure 1:**
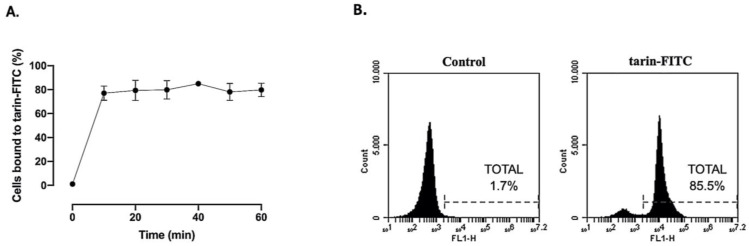
Tarin binding to hematopoietic bone marrow cells. (**A**) Tarin (25 µg/mL) labeled with FITC was added to murine BM cell suspension and the percentage of cells bound to tarin-FITC was monitored every 10 min up to 60 min by flow cytometry. (**B**) Representative histogram displaying the percentage of tarin-FITC-positive bone marrow (BM) cells (tarin-FITC panel) after 60 min and non-stained cells (control panel). Histograms are representative of three independent experiments.

An increase in the percentage of BM cells bound to tarin-FITC was detected early, after 10 min of incubation, ranging from 78% ± 7 to 86% ± 2 over time with no significant variation up to 60 min, indicating that tarin was able to bind to BM cells within the first 10 min and the interaction is sustained for at least 1 h, as determined by a flow cytometry analysis (Figure 1A,B). According to dot plots, tarin-FITC binding seems to exclude cell debris and erythrocytes, as demonstrated in Figure S1, right panel.

### 3.2. Tarin Stimulates Bone Marrow Cell Proliferation and Morphological Changes

**Figure 2:**
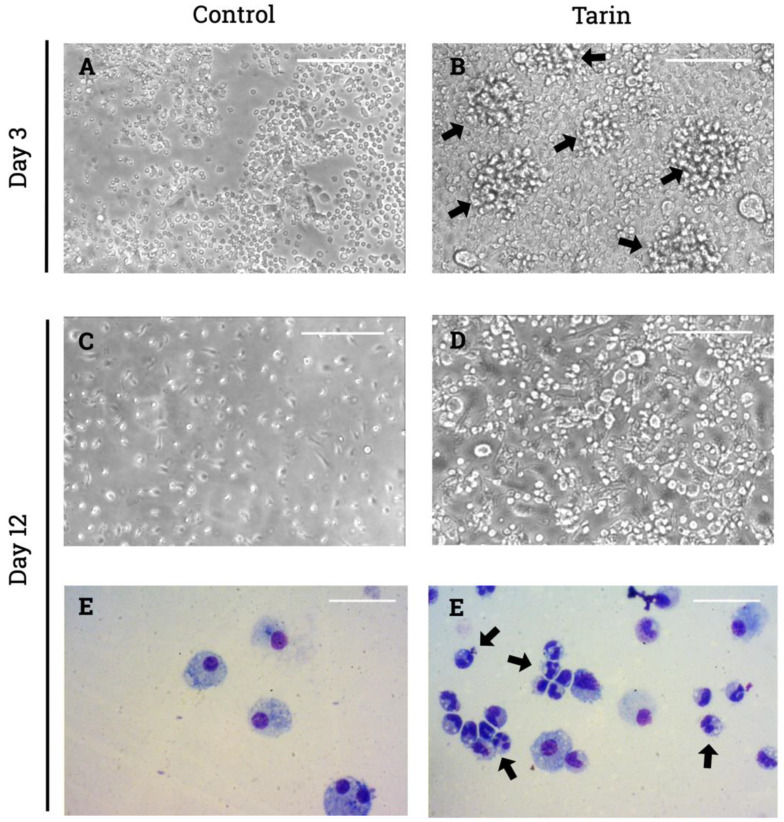
Representative photomicrographs of tarin-stimulated bone marrow cell cultures. Murine BM cells challenged with tarin at 25 µg/mL were cultured for 12 days and photomicrographs were acquired on the 3rd (**B**) and 12th (**D**,**F**) days post-stimulation. Photomicrographs of non-stimulated cultures cells were acquired on the 3rd day (**A**) and 12th day (**C**,**E**). Arrows indicate clusters/colonies (**B**) and neutrophils (**F**). Photomicrographs were acquired using an inverted-phase microscope at 200× (**A**–**D**) or a vertical microscope at 400× (**E**,**F**) magnification.

Tarin stimulated BM cell proliferation and differentiation in a dose-dependent manner. Increases in the number of clusters, colonies/field and in cell density (% occupied area/field) were observed on the 3rd and 12th culture days compared to controls (Table 1 and Figure 2).

Interestingly, on the 3rd culture day, adherent cells displaying fibroblastic morphology were more numerous in tarin-stimulated cultures (data not shown), becoming easily detectable on the 12th day. In parallel, large, rounded cells were observed, mainly at the highest tarin doses (Figure 2B,D). In non-stimulated cultures, both non-adherent and adherent cells were rare (Figure 2A,C). Cells stained by the May-Grunwald-Giemsa method revealed the prevalence of granulocytes (neutrophils) on the 12th culture day (Figure 2F), in contrast to control cultures, where these cells were absent (Figure 2E).

A cell density analysis calculated as the percentage of occupied area per field confirmed a pronounced proliferation by the 3rd day post-stimulation when bone marrow cells were exposed to tarin doses ranging from 6 to 100 μg/mL (Table 1). However, treatment with 6 μg/mL tarin also led to a non-sustainable increase in the occupied area, since a decrease was observed on the 12th day (Table 1). Similarly, an increase in the number of clusters and colonies/fields was observed on the 3rd day following tarin treatment at doses ≥12 μg/mL. However, this increase was sustained up to the 12th day only when doses ≥25 μg/mL were used (Table 1).

Based on these data, to obtain sustained effects, the bone marrow cell cultures must be exposed to at least 25 μg/mL tarin. Therefore, this was the amount adopted for the subsequent experiments.

### 3.3. Synergic Tarin and Growth Factor Effects on Bone Marrow Cell Cultures

**Figure 3:**
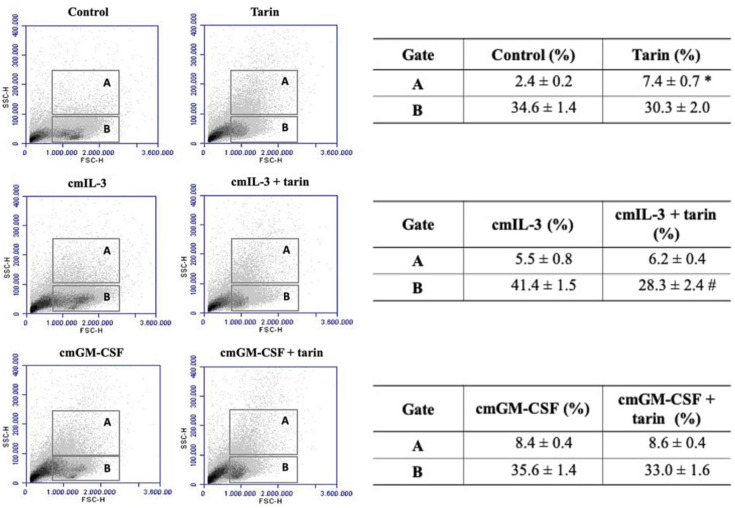
Effect of tarin and/or growth factors on the BM cell distribution profile during a 5 day-culture. Cells (2 × 10^6^ cells/mL) from C57BL/6 mice bone marrows were cultured in an RPMI-1640 media (control) or challenged with 10% (*v/v*) of a conditioned medium highly enriched with IL-3 (cmIL-3) or GM-CSF (cmGM-CSF) or with tarin at 25 µg/mL. A flow cytometry analysis was performed, and cell percentages were analyzed in gates A and B, determined according to cell granularity (SSC—side scatter). The presented dot plots are representative of three independent experiments (**left panel**) and are expressed as means ± SD of bone marrow cell percentages (**right panel**). * and ^#^ indicate the significance level * *p* < 0.0001 compared to the control, and ^#^ *p* < 0.0001 compared to cmIL-3. IL-3—Interleukin 3; GM-CSF—granulocyte/macrophage-colony-stimulating factor.

A representative dot plot demonstrated granularity variation (SSC) of murine BM cells cultured for 5 days in the presence of 25 µg/mL tarin, combined or not with a conditioned medium highly enriched with growth factors (cmIL-3 and cmGM-CSF) (Figure 3, left panel). Challenged cells were analyzed considering two specific gates, termed A and B regions, delimited according to their FSC × SSC parameters.

On the 5th day, the addition of 25 µg/mL tarin to BM cell culture resulted in a cell distribution profile modification, represented by an increase in the percentage of cells in gate A (7.4 ± 0.7%) when compared to the control group (2.4 ± 0.2%), indicating an increased granulocytic population. No alterations in gate B were observed upon tarin stimulation (30.3% ± 2.0) compared to the control (34.6% ± 1.4), although the distribution profile seems quite different (Figure 3, right panel).

A highly enriched conditioned medium containing IL-3 increased the cell percentage (5.5% ± 0.8) in gate A, similar to the observed in the combined cmIL-3 + tarin treatment (6.2% ± 0.4). The percentage of cells in gate B increased upon cmIL-3 stimulation (41.4% ± 1.5) compared to the control (34.6% ± 1.4) or tarin (30.3% ± 2.0). However, the combination of tarin and cmIL-3 decreased the percentage (28.3% ± 2.4) in gate B when compared to the cmIL-3-treated and control cultures (34.6 ± 1.4%) (Figure 3, right panel), but similar to tarin alone.

A highly enriched conditioned medium containing GM-CSF increased the cell percentage in gate A (8.4 ± 0.4%) similarly to tarin-treated culture (8.6% ± 0.4), when compared to the control. Stimulation of BM cells with cmGM-CSF + tarin did not induce a distinct response compared to cmGM-CSF or tarin challenges. The percentage of cells in gate B was not altered following cmGM-CSF treatment (35.6% ± 1.4) or cmGM-CSF + tarin-treated culture (33.0% ± 1.6) compared to the control, (Figure 3, right panel).

### 3.4. Tarin and/or Growth Factors Promote Phenotype Changes in BM Cells

Considering the morphological characteristics of lymphocytic and granulocytic cells, the frequency of myeloid Gr-1^+^ (Ly-6G/Ly-6C) cells was analyzed in gate A, while the frequency of CD3+ and CD19+ lymphocytic cells was analyzed in gate B of the dot plots (Table 2). When stimulated with tarin (25 µg/mL) for 5 days, the percentage of Gr-1^+^ BM cells increased significantly (23.3% ± 4.3), while the percentage of CD19^+^ cells decreased (21.1% ± 2.5) accompanied by an increase in CD3^+^ cells (48.3% ± 3.3) compared to their respective control cultures (Table 2).

To investigate whether the myeloid or lymphoid lineages were favored, the expression of IL-7Rα was investigated in cells within gate B (Table 3). Except for cmGM-CSF + tarin culture (26.5% ± 1.1), which maintained similar levels to the control culture (27.2% ± 2.1), the distinct cell stimulators, namely tarin and/or growth factors, led to a decrease in the percentage of IL-7Rα^+^ cells. This decrease was more pronounced (*p* < 0.0001) in the presence of tarin (9.7% ± 0.7) and cmIL-3 + tarin (11.4% ± 0.7) compared to the control culture (27.2% ± 2.1) (Table 3).

## 4. Discussion

Stimulation or suppression of immune system components have long been strategically targeted as preventive approaches or therapeutic treatments for several pathologies, such as cancer, diabetes, obesity, among other diseases [35,36,37,38,39]. Immunomodulatory molecules are abundantly found in food matrices ordinarily included in human diets. Taro corm is a tubercle traditionally consumed in Asia, West African countries, the USA, Canada, Japan, Turkey, and Central and South America countries, where it is considered and used as a food with medicinal effects to treat several physiopathological conditions through immune response stimulation to restore health status. Many studies have proven that taro corms contain several bioactive molecules, corroborating popular knowledge and its applied medicinal purposes [1,5,40,41]. Tarin is included among these bioactive molecules and has been extensively studied for over 15 years. Its structural and binding characteristics have been extensively investigated to better explore its potential for application as an antitumoral and immunomodulatory agent [1,8,14,15,17,23,24]. In the present study, in vitro tarin effects on myeloid and lymphocytic populations from mice BM were investigated, revealing an immunomodulatory impact on both lineages. In previous studies, tarin treatment promoted the maintenance of granulocytic progenitors and stimulated granulocyte repopulation, both in vitro and in vivo [15]. These findings were reinforced herein by the observed increases in cell density (% of occupied area/field) and in the number of clusters and colonies per field, which can be unequivocally visualized in cultures challenged with tarin in contrast to the control cultures, where a decrease in cell density or even an absence of clusters and colonies was observed by the 12th day of culture. According to previous studies, the 3rd day cultures are still populated by cells directedly isolated from mice BM. These cells cannot survive in the absence of stimulus, leading to rapid decreases in cell density, as observed herein, markedly visualized on the 5th culture day [15]. Tarin at ≥12 μg/mL prevents this decrease to some degree, and is able to induce an increase in cell density and clusters/colonies compared to the initial time point, suggesting a proliferative/differentiation and pro-survival effect, favoring the granulocytic lineage, especially neutrophils. Moreover, considering that cultures were challenged with a single dose, tarin exhibits a prolonged effect that persisted up to the 12th day, indicating the possibility that the effect could be strengthened by multiple or increased dosages. This sustained response probably results in tarin progenitor cell protection, followed by BM repopulation through proliferative activity and differentiation. It is also possible that these effects are a result of the establishment of a feeder monolayer constituted by stromal cells. The combination of both mechanisms should also be considered.

In adults, hematopoiesis occurs mainly in the bone marrow, from a common progenitor (stem cell) that exhibits self-renewal and totipotent ability, originating all hematopoietic lineages. Hematopoiesis is governed by external stimuli, such as cell-cell contact and cytokine signaling. Stromal cells play a critical role in this process by releasing cytokines, including survival factors, colony-stimulating factors, interleukins, and by promoting stimulation through cell contact [42,43,44]. BM cells challenged with tarin for 12 days were not only able to proliferate but, in fact, favored the establishment of a confluent monolayer of fibroblast-like cells, a morphological characteristic of stromal cells, which could also explain hematopoietic cells sustenance in culture, an effect not observed in the absence of a tarin stimulus (control group). Further studies using molecular markers are essential to confirm the presence of stromal cells in tarin-stimulated cultures and to determine tarin interactions, triggering the release of cytokines, survival, or growth factors, resulting in the biological responses described in this study.

Besides the hypothetical activation of stromal cells, leukocytes can also be stimulated by tarin, triggering the expression of cytokine genes like IL-2, IL1β, INF-γ, and TNF-α, as previously demonstrated by the addition of tarin to mice splenocytes, suggesting that the observed in vitro effects could also be a consequence of the cytokines released by tarin-stimulated hematopoietic cells [7]. Culture supernatants may be analyzed to investigate this possibility.

Moreover, tarin seems to exhibit a cytokine-mimetic effect similar to IL-3 and GM-CSF, with granulocytic lineage cell stimulation. Hematopoietic stem cells and multipotent progenitors are more sensitive to IL-3, while committed myeloid progenitors are sensitive to GM-CSF signaling as they are able to originate cells from the myeloid lineage [45,46,47]. Not surprisingly, both cmIL-3 and cmGM-CSF-stimulated cultures promoted an increase in the percentage of cells that reside in the granulocytic gate (A region of the dot plot) on the 5th day. Tarin also affected cells in the granulocytic gate, increasing the cell percentage to levels higher than cmIL-3, which promoted a discrete response by itself. The fact that cmIL-3 when combined to tarin led to a similar effect to that of tarin alone suggests a non-antagonistic effect and the possible prevalent action of tarin over cytokines, since all tarin group dot plots exhibited the same cell distribution shape. A flow cytometry analysis confirmed the influence of tarin on the myeloid population displaying a Gr-1^+^ (Ly6-C/Ly6-G) phenotype, abundantly expressed in granulocytes, immature and mature myeloid cells, including monocytes/macrophages, and rarely in lymphocytes or non-compromised progenitors [48,49]. Photomicrographs of tarin-stimulated cell cultures reinforce the fact that tarin affects granulocytic lineages, especially neutrophils. Both GM-CSF and IL-3 have been used for clinical purposes to prevent immunosuppression or reestablish the immunological status of individuals under chemotherapy or drug-induced immune depression [39]. Similarly, our previous experimental results have shown that tarin exerts similar in vivo effects. The administration of tarin to cyclophosphamide-immunosuppressed mice was able to minimize drug-induced leukopenia, with a faster recovery of peripheral leukocyte numbers and the prevention of erythrocyte progenitor death, which can be explained by the in vitro data described herein [15]. Tarin may, thus, be used as a supportive therapy for diseases and treatments such as chemo and radiotherapies to aid in the recovery of immune system homeostasis without causing the side effects usually associated with current drugs [50,51,52].

The percentage of cells in gate B of the dot plots, corresponding to the lymphocytic lineage, was not altered upon tarin treatment in the cell culture, although the cell population distribution differed from the control group. Albeit cmIL-3 alone promoted an increase in cells in B region, when cmIL-3 and tarin were combined, the percentage of cells decreased under the control levels but similarly to tarin alone. The discrepant increase in the cell percentage in gate B produced by cmIL-3 is an expected result, since this cytokine is able to act on a wide range of hematopoietic cells, including non-lineage-restricted progenitors, which includes cells from both regions, as mentioned previously. Again, tarin effects seem to prevail over cmIL-3 when they are combined, considering that all tarin groups exhibited the same cell population distribution profile. A detailed investigation on these cell types indicated a high increase in the percentage of CD3^+^ cells (T cell lineage) alongside a decrease in CD19^+^ cells (B cell lineage) within gate B. Both molecular markers, CD3 and CD19, are known as lineage-restricted and found in progenitors and mature and immature lymphocytes, suggesting that tarin may lead to a biased effect upon lymphopoiesis, favoring T cell development over B lymphocytes in the bone marrow [53,54]. Similarly, previous studies have shown that the intraperitoneal inoculation of crude taro extracts induced a transient bone marrow decrease in the number of immature (B220^+^IgM^−^) and mature (B220^+^IgM^+^) B lymphocytes on day 5 post-treatment in C57BL/6 mice. This was followed by an increase in immature B lymphocytes 10 days after stimulation. On the other hand, tarin increased the proliferation of B220^+^ cells in vitro and in vivo in the spleen [14,17]. Further studies should be conducted to understand the dynamics of differential tarin effect on B lymphocytes in primary and secondary organs.

To understand the stimulatory action of tarin on B and T lymphocyte populations, IL-7Rα expression was evaluated within gate B and revealed that the modifications in CD19 and CD3 molecular markers was accompanied by a decrease in the percentage of IL-7Rα^+^ cells in tarin-stimulated cultures and in cultures treated by cmIL-3 and/or cmGM-CSF. The IL-7 receptor is an important molecule for parental differentiation between lymphoid and myeloid strains. The presence of IL-7Rα characterizes lymphoid lineage progenitors, while its absence leads to myeloid lineage progenitor differentiation [46,47]. IL-7Rα expression is dynamically up- and downregulated according to lymphopoiesis stage. It is found in CD19^+^ cells from the pro-B stage, where it is highly expressed, becoming subsequently downregulated until cell progenitor pre-B stage and is absent in later B cell development stages. In T cells lineage, IL-7Rα is highly present in CD3- progenitors and downregulated throughout development, reaching low expression in CD3+ progenitors and becoming present in subsequent stages. Moreover, although not yet well understood, IL-7Rα expression in mature CD3+ T lymphocytes can be further up- or downregulated according to IL-7 availability or TCR (T-cell receptor) stimulation [53,55,56]. These findings may explain the increase in the CD3 marker with a concomitant decrease in IL-7Rα in gate B upon tarin treatment. After 5 days, tarin may preferentially stimulate T lymphopoiesis until it reaches a stage where IL-7Rα expression is diminished. Effects of taro corm molecules on T cell populations have been demonstrated by in vitro stimulation of murine splenocytes using a soluble extract obtained from poi, a pasty preparation from cooked taro corms, reinforcing the data obtained herein [57].

It has been previously reported that tarin is capable of interacting with mannose-based and complex N-glycans, especially in the Lewis Y (CD174) and H2 (CD173) antigens, with low or no binding affinity to free mannose [23]. These antigens are found in CD34^+^ hematopoietic progenitor cells, reinforcing the possibility of progenitor protection following tarin binding. Corroborating the data from the present study, N-glycans and the expression of CD34 on the surface of hematopoietic progenitor cells characterize a heterogeneous population displaying the multipotent ability to reconstitute both the myeloid and lymphoid systems in a supra-lethally irradiated host [58,59,60]. However, BM cell suspension incubated with tarin-FITC did not display a cell-type preference binding pattern, indicating multi-specificity, except for erythrocytes, as suggested by the dot plot of tarin-FITC-negative cells. Since these data correspond to preliminary studies, a detailed investigation should be performed. Coincidently, CD45 molecular marker is present in the majority of hematopoietic cells but not in erythrocytes and plasmatic cells [61]. However, further studies applying molecular markers are necessary to determine if tarin interacts with CD45 and/or CD34 in this condition. The rapid and broad binding of tarin to hemopoietic cells and, possibly, to the multipotent stromal cell population may create a special microenvironment for lymphoid and hemopoietic cell proliferation and differentiation.

Although tarin effects on the bone marrow stroma were not evaluated, nor a more robust phenotypic analysis, the results presented herein undoubtedly indicate the immunomodulatory potential of this taro lectin. Since tarin mechanisms of action have not yet been elucidated, additional studies should be performed to evaluate if tarin acts directly on the surface of hematopoietic cells, intracellularly, or both, stimulating proliferation/differentiation or cytokine release. Additionally, the observed responses could also be the result of tarin interaction with stromal bone marrow cells with consequent cytokine release or the combination of both.

## 5. Conclusions

Taro corms exhibit not only nutritional importance but also potential pharmacological activities that may be explored as functional food fortification with preventive or healing properties, or as a source of medicinal molecules to treat several pathophysiological conditions. Although the tarin mechanism of action is not clear, lectin exhibited immunomodulatory potential, displaying cytokine-mimetic effects able to stimulate mice BM hematopoietic cell proliferation/differentiation, preferentially the granulocytic and T lymphocytic lineages, and potential progenitor cell protection, facilitating stroma establishment followed by cell culture repopulation. The findings described herein, combined with previous data, support the statement that tarin displays a latent chemotherapeutic adjuvant potential that deserves to be further explored in clinical trials.

## Figures and Tables

**Table 1:** Dynamic response of bone marrow cells treated with tarin.

Experimental Condition	Tarin Stimulus (µg/mL)	% Occupied Area/Field ^1^		Number of Clusters and Colonies/Field	
		Day 3	Day 12	Day 3	Day 12
**Control**	-	51.2 ± 5.2	31.6 ± 3.5	2.5 ± 2.4	0.0 ± 0.0
**Tarin**	6.0	68.0 ± 2.3 *	26.8 ± 3.7	4.5 ± 2.1	1.8 ± 0.1
	12.0	63.9 ± 2.7 *	44.1 ± 2.9 *	13.8 ± 2.2 **	3.3 ± 1.0
	25.0	64.4 ± 2.3 *	84.2 ± 3.2 *	17.8 ± 3.3 *	17.5 ± 3.4 *
	50.0	64.1 ± 3.1*	85.2 ± 3.2 *	28.5 ± 4.2 *	19.8 ± 5.0 *
	100.0	78.4 ± 2.6 *	91.4 ± 3.7 *	29.5 ± 3.9 *	18.3 ± 2.6 *

Bone marrow cells culture stimulated or not with tarin (6–100 µg/mL) were evaluated on the 3rd and 12th culture days. ^1^ Percentage of the cell-occupied area occupied in each analyzed field under microscope visualization. Values are expressed as the means ± SD of three independent experiments carried out in duplicate. Significant differences at significance levels of * *p* < 0.0001 and ** *p* < 0.001 compared to control.

**Table 2:** Percentage of Gr-1^+^, CD3^+^, and CD19^+^ tarin-stimulated bone marrow cells on the 5th day.

Gate	Cell Phenotype	Control (%)	Tarin (%)
**A**	**GR-1^+^**	8.8 ± 2.5	23.3 ± 4.3 ***
**B**	**CD3^+^**	19.6 ± 1.4	48.3 ± 3.3 **
	**CD19^+^**	32.5 ± 2.5	21.1 ± 2.5 **

Values indicate the percentage of Gr-1^+^, CD3^+^, and CD19^+^ bone marrow cells in gates A or B after 5 days of treatment or not with tarin at 25 µg/mL. Values are expressed as the means ± SD of three independent experiments carried out in duplicate. Significant differences at ** *p* < 0.001 and *** *p* < 0.01 compared to the control.

**Table 3:** Percentage of IL-7Rα^+^ bone marrow cells stimulated with tarin and/or growth factors for 5 days.

Stimulus	Gate B (%)
Control	27.2 ± 2.1 ^a^
Tarin	9.7 ± 0.7 * ^b^
cmIL-3	21.1 ± 1.3 ^c^
cmIL-3 + tarin	11.4 ± 0.7 ^# b^
cmGM-CSF	22.7 ± 0.3 ^c^
cmGM-CSF + tarin	26.5 ± 1.1 ^a^

Values indicate the percentage of IL-7R𝛼^+^ bone marrow cells in gates A or B after 5 days of treatment or not with tarin at 25 µg/mL and/or 10% (*v/v*) conditioned medium highly enriched with IL-3 (cmIL-3) or GM-CSF (cmGM-CSF). Values are expressed as the means ± SD of three independent experiments carried out in duplicate. * and ^#^ indicate the significance level * *p* < 0.0001 compared to control, ^#^ *p* < 0.0001 compared to cmIL-3. Distinct letters (a–c) denote significant differences at *p* < 0.05. IL-3—interleukin 3; GM-CSF—granulocyte/macrophage colony-stimulating factor.

## Data Availability

The data presented in this study are available in the article or Supplementary Materials.

## Supplementary Materials

The following is available online at https://www.mdpi.com/1999-4923/13/3/350/s1, Figure S1: Tarin binding to hematopoietic bone marrow cells.
