# Supplementary material for: Taro Lectin Can Act as a Cytokine-Mimetic Compound, Stimulating Myeloid and T Lymphocyte Lineages and Protecting Progenitors in Murine Bone Marrow
Source: Pharmaceutics. 2021 Mar 7;13(3):350. doi: 10.3390/pharmaceutics13030350 (PMC8001523; doi:10.3390/pharmaceutics13030350)
Supplement: Supplementary file 1 [file pharmaceutics-13-00350-s001.pdf]

pharmaceutics

# Supplementary Materials: Taro Lectin Can Act as a Cytokine-Mimetic Compound, Stimulating Myeloid and T Lymphocyte Lineages and Protecting Progenitors in Murine Bone Marrow

Erika Bertozzi de Aquino Mattos, Patricia Ribeiro Pereira, Lyris Anunciata Demétrio Mérida, Anna Carolina Nitzsche Teixeira Fernandes Corrêa, Maria Paula Vigna Freire, Vania Margaret Flosi Paschoalin, Gerlinde Agate Platais Brasil Teixeira, Maria de Fátima Brandão Pinho and Maurício Afonso Verícimo

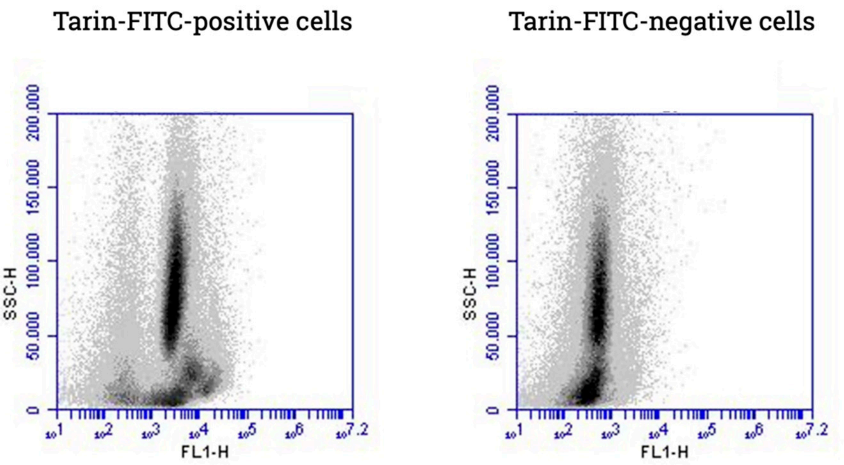


**Figure S1.** Tarin binding to hematopoietic bone marrow cells. Tarin (25 µg/mL) labeled with FITC was added to murine BM cell suspension and the percentage of cells bound to tarin-FITC was monitored by flow cytometry every 10 min up to 60 min. Representative dot plots display the distribution profile of tarin-FITC-positive cells (stained BM cells) (*left panel*) and tarin-FITC-negative cells (non-stained BM cells) (*right panel*) after 30 min. Dot plots are representative of three independent experiments.
